# *Drosophila* expressing mutant human *KCNT1* transgenes make an effective tool for targeted drug screening in a whole animal model of KCNT1-epilepsy

**DOI:** 10.1038/s41598-024-53588-x

**Published:** 2024-02-09

**Authors:** Rashid Hussain, Chiao Xin Lim, Zeeshan Shaukat, Anowarul Islam, Emily A. Caseley, Jonathan D. Lippiat, Grigori Y. Rychkov, Michael G. Ricos, Leanne M. Dibbens

**Affiliations:** 1 Epilepsy Research Group, Clinical and Health Sciences, Australian Centre for Precision Health, University of South Australia, Adelaide, SA 5000 Australia; 2 Pharmacy, School of Health and Biomedical Sciences, RMIT University, Bundoora, VIC 3083 Australia; 3 College of Medicine and Public Health, Flinders University, Bedford Park, SA 5042 Australia; 4 School of Biomedical Sciences, Faculty of Biological Sciences, University of Leeds, Leeds, LS2 9JT UK; 5 School of Biomedicine, University of Adelaide, Adelaide, SA 5005 Australia; 6 South Australian Health and Medical Research Institute, Adelaide, SA 5005 Australia

**Keywords:** Ion channels in the nervous system, Epilepsy

## Abstract

Mutations in the *KCNT1* potassium channel cause severe forms of epilepsy which are poorly controlled with current treatments. In vitro studies have shown that *KCNT1-*epilepsy mutations are gain of function, significantly increasing K^+^ current amplitudes. To investigate if *Drosophila* can be used to model human *KCNT1* epilepsy*,* we generated *Drosophila melanogaster* lines carrying human *KCNT1* with the patient mutation G288S, R398Q or R928C. Expression of each mutant channel in GABAergic neurons gave a seizure phenotype which responded either positively or negatively to 5 frontline epilepsy drugs most commonly administered to patients with *KCNT1*-epilepsy, often with little or no improvement of seizures. Cannabidiol showed the greatest reduction of the seizure phenotype while some drugs increased the seizure phenotype. Our study shows that *Drosophila* has the potential to model human *KCNT1*- epilepsy and can be used as a tool to assess new treatments for *KCNT1*- epilepsy.

## Introduction

Mutations in *KCNT1* have been identified in a range of epilepsies with drug-resistant seizures^1^. *KCNT1* mutations are the major cause of epilepsy of infancy with migrating focal seizures (EIMFS)^1–3^, where cognitive and developmental regression follow seizure onset. *KCNT1* mutations also cause other severe epilepsies beginning in infancy, including West Syndrome and Otahara Syndrome^1^. They also contribute to a range of focal epilepsies, which can have a later age of onset (in childhood or adolescence), including sleep-related hypermotor epilepsy^1,4,5^. *KCNT1*-epilepsy is often debilitating and while treatment with anti-epilepsy drugs can reduce seizures in some patients, seizure suppression is usually incomplete and the development of new treatments is needed^3^.

*KCNT1* encodes an ion channel which is a major contributor of the sodium-activated potassium Ik_Na_ current which down regulates neuronal excitability. Following a rise in intracellular [Na^+^], KCNT1 channels are thought to increase K^+^ current and prolong the slow afterhyperpolarisation phase following an action potential, thereby reducing the chance of repetitive neuronal firing^1,6,7^. Almost all *KCNT1* epilepsy mutations are heterozygous and missense, predicted to change a single amino acid in the protein^3^ and de novo mutations most often account for severe cases. All mutations analysed in vitro to date (apart from T314A^8^) significantly increase K^+^ currents in comparison to normal KCNT1 channels^8–12^. There is evidence suggesting that increased K^+^ currents in inhibitory neurons may be associated with *KCNT1*-related seizures^8,13,14^. Reducing the activity of inhibitory neurons may explain how neuronal hyperexcitability, the mechanism underlying seizures, occurs in *KCNT1*-epilepsy.

There are currently no highly effective treatments for *KCNT1*-epilepsy and no drugs available that specifically target the KCNT1 channel. Demonstration that increased K^+^ current due to overactivity of the KCNT1 channel is associated with seizures suggests that reducing or blocking KCNT1 channel activity may inhibit seizures, and this has directed new drug screening efforts^15^. Currently, anti-epileptic drugs including carbamazepine, vigabatrin and valproic acid, as well as cannabidiol (CBD) are frontline medications commonly administered for *KCNT1-*epilepsy^3,16–18^. However, even combinations of multiple drugs (sometimes eight or more) usually fail to suppress seizures, with some patients continuing to experience up to one hundred seizures per day^3^. In vitro studies have shown that the ion channel blocker quinidine reduces the increased current amplitude produced by *KCNT1*-epilepsy mutations^9,11,19^. However, it has shown variable results in patients and can have serious side effects^3,19–21^.

**Figure 1:**
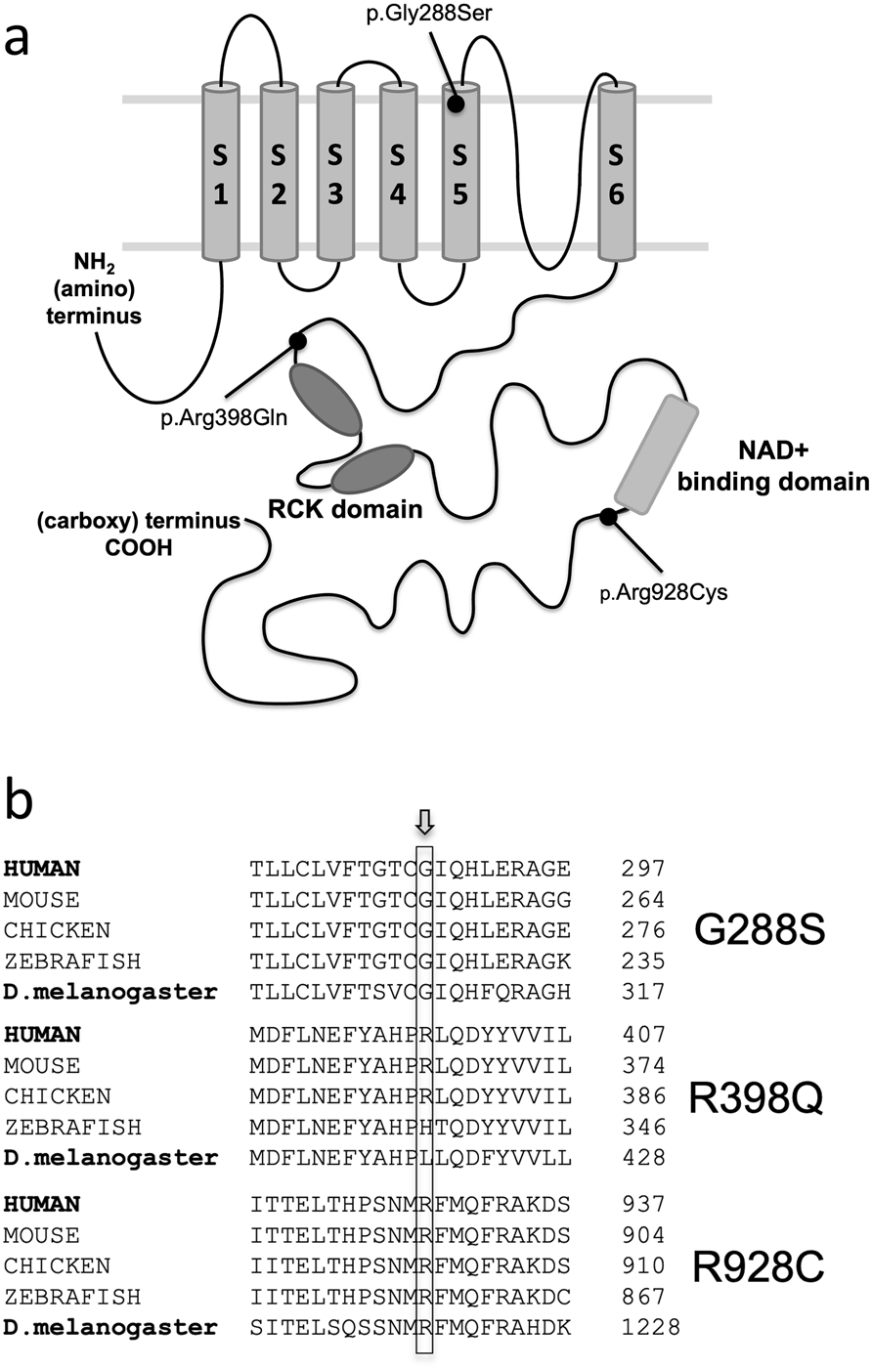
Position and evolutionary conservation of mutated amino acid residues. Position and evolutionary conservation of the amino acid residues altered by the three patient *KCNT1* missense mutations investigated in this study, G288S, R398Q and R928C**. (a)** Schematic diagram of the KCNT1 channel showing the positions of the three mutations investigated in this study. **(b)** Alignment of the orthologous KCNT1 proteins found in different species showing the high evolutionary conservation of the amino acids altered by the 3 *KCNT1* mutations investigated in this study.

In this study we sought to investigate if *Drosophila* could be used to model *KCNT1*-epilepsy and if the seizure phenotype responded to some of the drugs currently used to treat patients. Many of the genes and pathways identified in human epilepsy are highly conserved in *Drosophila*^22^. The animal has been used to model other forms of human epilepsy, including Dravet Syndrome and Generalised Epilepsy with Febrile Seizures Plus (GEFS+) due to mutations in the sodium channel gene *SCN1A*^23,24^ and can make powerful tools for therapeutic screens^25^. The majority of *KCNT1*-epilepsy mutations cluster around three, highly conserved, functional domains in the KCNT1 protein^1^. A mutation from each region was selected to be analysed: c.862G > A, p.G288S in the S5 segment of the pore domain, c.1193G > A, p. R398Q in the RCK1 (Regulator of K^+^ Conductance) domain and c.2782C > T, p.R928C adjacent to the NAD^+^ binding domain (Fig. 1). G288S and R398Q mutations have been identified in patients with a range of epilepsy phenotypes, ranging from very severe infantile onset epilepsies to less severe and later onset focal epilepsies, whereas R928C mutations are only associated with the latter. The three mutations are recurrently found in patients, collectively accounting for approximately 20% of cases^1,3^. Thus, successful modelling of human *KCNT1*-epilepsy with these mutations would be relevant for a significant proportion of patients.

To generate the *Drosophila* models, human *KCNT1* with the patient mutation G288S, R398Q or R928C was introduced into *Drosophila* by transgenesis. The mutant KCNT1 channels were expressed in different neural tissues, including pan-neural, glial, excitatory (cholinergic) neurons and inhibitory (GABAergic) neurons, and investigated for a seizure phenotype using bang sensitive behavioural assays^26,27^. To investigate the potential of the *Drosophila* models in assessing treatments for patients with *KCNT1* epilepsy, five of the frontline anti-epilepsy drugs currently administered to patients with KCNT1 epilepsy which have been shown to reduce, but rarely stop, seizures in some patients were analysed for their effects on the seizure phenotype in our *Drosophila* models.

## Results

### Investigation of a seizure phenotype in *Drosophila* with G288S, R398Q or R928C mutant KCNT1

To investigate if *Drosophila c*arrying *KCNT1* mutations showed a seizure phenotype, three transgenic lines were generated with mutated human *KCNT1* (Fig. 1a,b). Each *Drosophila* line carried a human *KCNT1* transgene with a heterozygous missense mutation that has been identified in patients, G288S, R398Q or R928C. Wild type (WT), normal, human *KCNT1* (NM_020822.3) was used as a control. The UAS-GAL4 expression system was used to drive overexpression of the human *KCNT1* transgenes^28^. Each *Drosophila* line contained an upstream activating sequence (UAS) positioned before the *KCNT1* transgene. Genetic crosses were used to introduce the GAL4 transcription factor under the control of selected promoters, to drive expression of the WT or mutant KCNT1 channels in different tissues.

The KCNT1 channel is found in a wide variety of neurons in mammals^29,30^ and we expressed them in a range of neural tissue types in the *Drosophila* models. We first tried to see if driving expression of the mutant transgenes in all neurons using a pan-neural GAL4 driver could give a seizure phenotype. Using the pan-neural promoter *elav*^*C155*^*-GAL4* to drive mutant *KCNT1* expression in all neurons did not give any progeny and was embryonic lethal, thus a seizure phenotype was not able to be investigated (Table 1). Expression of WT human *KCNT1* in all neurons with *elav*^*C155*^*-GAL4* produced living progeny indicating overexpression of the WT human KCNT1 channel was not lethal. We then tried to express mutant human *KCNT1* transgenes in smaller subsets of neural tissues to see if they could give a seizure phenotype. First, the three mutant and one WT transgenes were expressed in excitatory neurons with the *CHAT-GAL4* driver containing the *Choline-Acetyltransferase* (*Chat*) gene promoter to drive expression in excitatory cholinergic neurons^31^. As was observed for pan-neural expression, only expression of WT *KCNT1* in cholinergic neurons gave living adult flies and expression of the three mutant human *KCNT1* lines was lethal (Table 1). The effects of expressing WT and mutant human *KCNT1* transgenes in glia, were assessed using the *reversed polarity* (*Repo)-GAL4* driver^32^. None of the 3 mutants gave any surviving progeny, and as before, the WT human KCNT1 expressed in glia gave viable offspring (Table 1). Interestingly, none of the surviving WT human *KCNT1* expressing flies with either *ELAV-GAL4*, *CHAT-GAL4* or *REPO-GAL4* displayed any seizure phenotype in bang-sensitive assays (Supplementary Fig. 1).

**Table 1:** Effects of wild type (WT) and mutant human KCNT1 transgene expression.

Mutant	Driver			
	Pan-neuronal Elav-GAL4	Cholinergic Chat-Gal4	GABAergic GAD1-GAL4	Glial Repo-GAL4
UAS-KCNT1-WT	Viable—no effect	Viable—no effect	Viable—no effect	Viable—no effect
UAS-KCNT1-G288S	Embryonic lethal	Embryonic lethal	Viable—bang sensitive	Embryonic lethal
UAS-KCNT1-R398Q	Embryonic lethal	Embryonic lethal	Viable—bang sensitive	Embryonic lethal
UAS-KCNT1-R928C	Embryonic lethal	Embryonic lethal	Viable—bang sensitive	Embryonic lethal

It has been postulated from experiments in cells and mice that one possible mechanism of seizure genesis may be due to the inhibition of inhibitory GABAergic interneurons^14^. As in humans, the major inhibitory neurotransmitter in the *Drosophila* central nervous system is γ-aminobutyric acid (GABA)^26^. Previous studies have suggested that reduced activity of inhibitory GABAergic neurons may be associated with seizures^8,13,14^, so we next expressed the WT and mutant human *KCNT1* transgenes in GABAergic neurons using the *GAD1-GAL4* line which drives GAL4 expression from a 3.089 kb fragment of the GAD1 promoter in GABAergic neurons^33–38^. Unlike, glial, pan-neural and excitatory neuron expression, *GAD1-GAL4* driven expression of the WT and three mutant human *KCNT1* transgenes gave surviving adults which were healthy and lived long enough to be investigated for a seizure phenotype in bang-sensitive behavioural assays^26,27^.

To quantify the effects on viability of driver and transgene combinations, the heterozygous (Driver/Balancer) *Gad1-Gal4/CyO* flies were crossed with WT human *KCNT1* and the three human *KCNT1* mutants. The average viable count of progeny with a given driver expressing human *KCNT1* (*Gad1-Gal4: UAS-human-KCNT1*) versus progeny with no-driver (*CyO: UAS-human-KCNT1*) was calculated and gave a viability ratio between 0.8 and 1. This suggested that the expression of WT human *KCNT1* or mutant human *KCNT1* using *GAD1-GAL4* at 24 °C showed minimal or no effect on the survival of offspring. In contrast, viability ratio analysis using the balanced drivers *CHAT-GAL4/CyO* and *REPO-GAL4/Tm3* for expressing any of the three mutant human *KCNT1* transgenes was less than 0.01 at 24 °C whereas expression of WT human *KCNT1* transgenes was close to 1.

**Figure 2:**
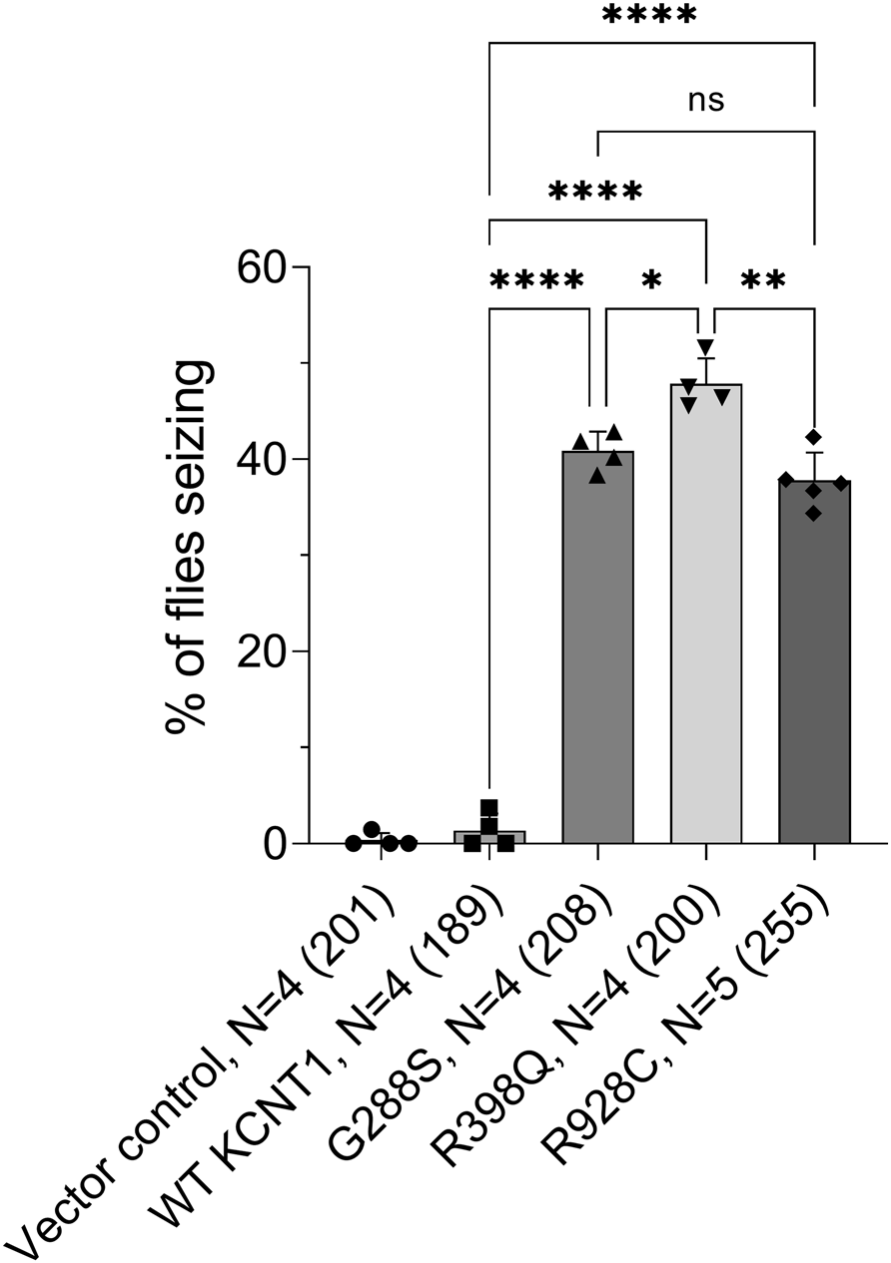
Expressing human *KCNT1* mutants in GABAergic neurons of *Drosophila* gives rise to seizures in a bang sensitive behavioural assay. *Drosophila* with the vector control and *Drosophila* expressing either WT or G288S, R398Q or R928C mutant human *KCNT1* in GABAergic neurons were analysed in the bang sensitive behavioural seizure assay. Percentage of *Drosophila* showing a seizure phenotype are shown for each line. N, is the number of independent crosses, with total number of flies in all crosses shown in brackets. The data were analysed using Brown-Forsythe and Welsh’s one-way ANOVA followed by Dunnett's T3 multiple comparisons test; ****P < 0.0001, ***P = 0.0003, **P = 0.0080, *P = 0.0411, ns—no significant difference.

As only the *GAD1-GAL4* driver gave any viable adult *Drosophila*, when expressing the mutant *KCNT1* transgenes, only this driver was able to be used for all future experiments on seizure analysis and drug rescue of seizures. We performed the ‘bang sensitive behavioural assay’ on *Drosophila* expressing the three human *KCNT1* patient mutations or WT human *KCNT1* in GABAergic neurons and calculated the percentage of animals showing a seizure phenotype for each genotype. The mutant *KCNT1* lines G288S, R398Q and R928C each showed a statistically significant seizure phenotype, while expression of WT human *KCNT1* in GABAergic neurons did not (Fig. 2). R398Q gave the strongest seizure phenotype with 48% of animals showing seizure activity followed by G288S with 41% and R928C with 38%.

### In vitro effects of currently used drugs on KCNT1 channels

**Figure 3:**
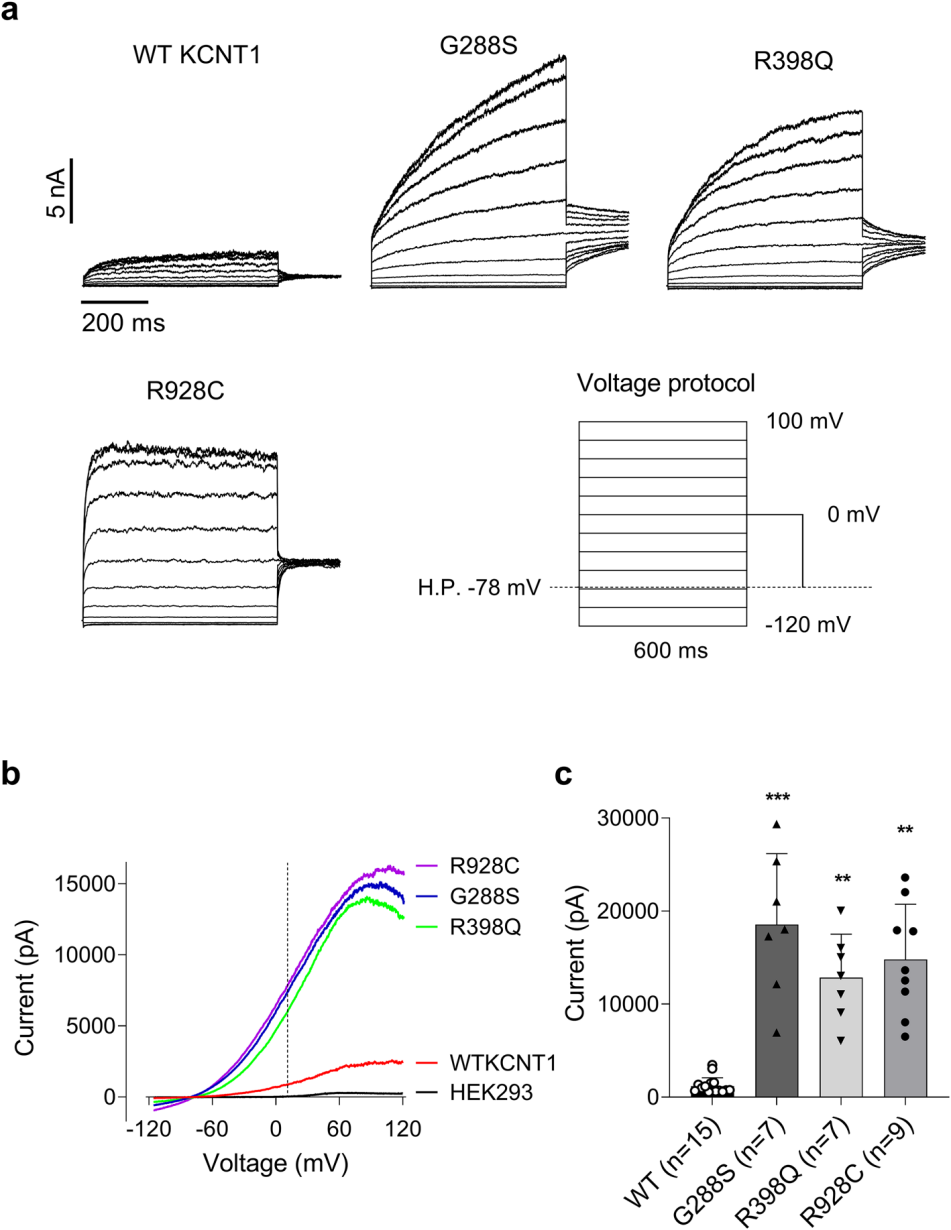
WT and mutant KCNT1 currents recorded in HEK293T cells. (**a**) KCNT1 currents recorded in HEK293T cells in response to the voltage steps ranging from − 120 mV to 80 mV in 20 mV increments followed by a voltage step to 0 mV. (**b)** Examples of the I-V plots of WT and mutant KCNT1 currents recorded in response to 100 ms voltage ramps between − 120 and 120 mV. For comparison, an example of the I-V plot recorded in non-transfected HEK293T cell is shown. The vertical dash line corresponds to 10 mV. (**c)** The average amplitudes of the WT and mutant KCNT1 currents measured at 10 mV using the I–V plots recorded in response to the voltage ramps between − 120 and 120 mV, similar to those shown in panel (**b).** Brown-Forsythe and Welsh’s one-way ANOVA followed by Dunnett's multiple comparisons test indicated that currents produced by the mutant constructs were significantly larger, compared to WT KCNT1 (*P* = 0.0003 (G288S); *P* = 0.0017 (R398Q) and *P* = 0.0026 (R928C)).

Having shown that expression of G228S, R398Q and R928C mutant *KCNT1* in GABAergic neurons gives a seizure phenotype, we next investigated if the phenotype generated by each of the three mutations responded to some of the drugs most commonly used to reduce seizures in patients with *KCNT1*-epilepsy. In the initial experiments, we looked at the in vitro effects of the drugs on KCNT1 channels in human cells using a HEK293T cell expression system and patch clamping analysis. Consistent with previous findings, the amplitudes of KCNT1 currents in cells expressing the G288S, R398Q and R928C mutants were significantly larger than those expressing WT KCNT1 (Fig. 3)^8^. Furthermore, the kinetics of KCNT1 currents were affected by the mutations as shown and discussed in our previous publication^8^.

**Figure 4:**
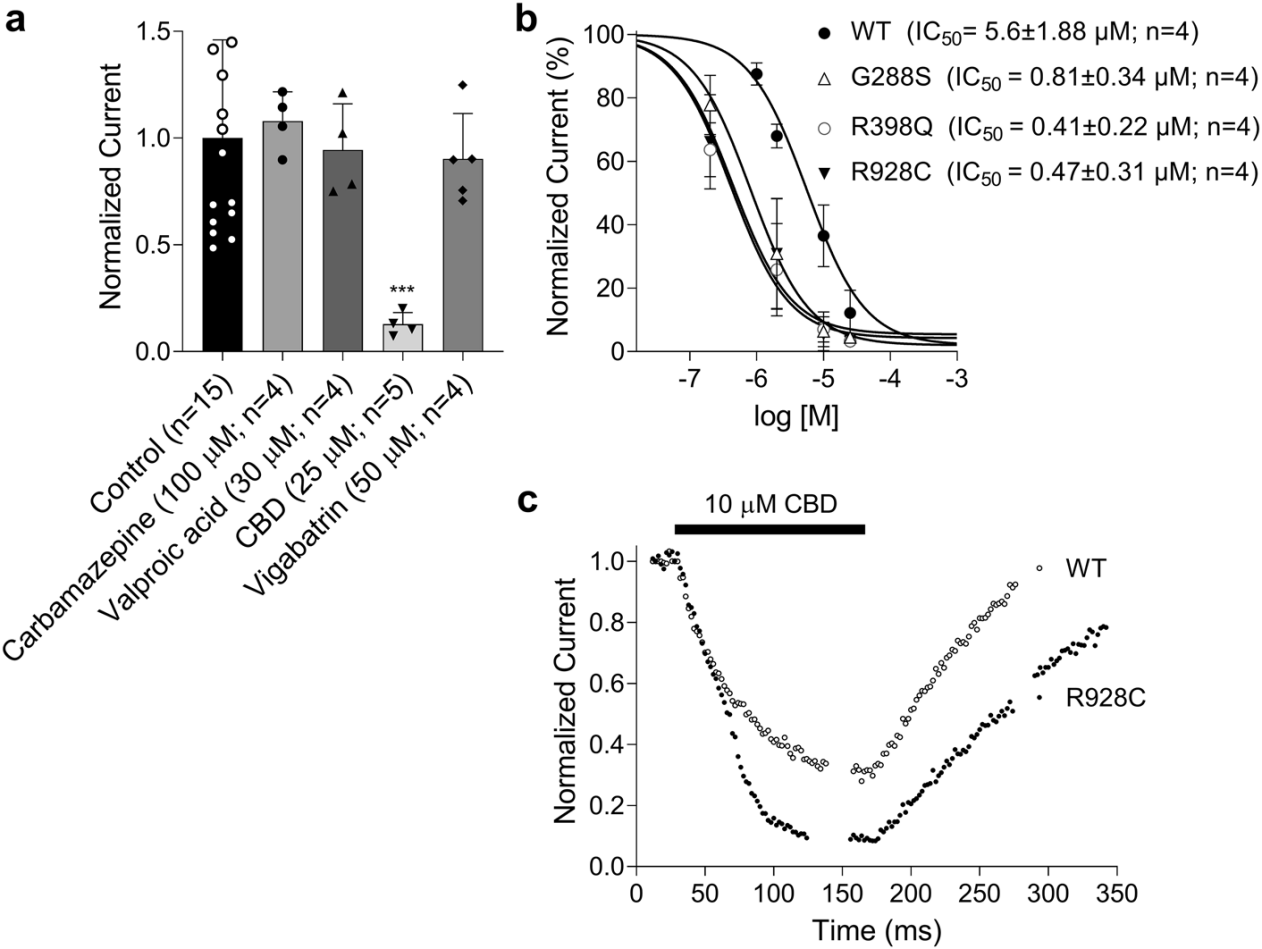
The in vitro effects of drugs on KCNT1 channels. (**a**) The average normalised WT KCNT1 current amplitude measured at 10 mV using I–V plots similar to those shown in Fig. 3b in the presence of the drugs. (**b)** The dose dependent inhibition of the WT and mutant KCNT1 channels by CBD. The curves represent the fit of the Hill equation (Eq. 1, Methods) to the experimental data. (**c)** The time course of KCNT1 inhibition by 10 µM CBD followed by the washout. Each point represents current amplitude measured at 10 mV from the I–V plots (see Fig. 3b) in response to the voltage ramps applied every 2 s.

Next, we analysed the effects of carbamazepine, valproic acid, vigabatrin and cannabidiol (CBD). The in vitro effects of quinidine were not investigated as part of this study as these have been published previously^9,11^. Each drug was first tested on WT KCNT1 channels at a range of concentrations, including well above their currently accepted potential doses for therapeutic use. The drugs were applied to the bath through the perfusion system and the amplitude of WT KCNT1 current was monitored by applying voltage ramps between − 120 and 120 mV every 2 s. Carbamazepine (100 µM), valproic acid (30 µM), and vigabatrin (50 µM) showed no effect on the current amplitude produced by WT KCNT1 within 5–10 min of application (Fig. 4a). Since the drugs showed no effects on the WT KCNT1 channels we did not test their in vitro effects on the mutant channels. In contrast, CBD (25 µM) inhibited almost 90% of the WT KCNT1 current in HEK293T cells (Fig. 4a). Addition of CBD to HEK293T cells expressing each of the KCNT1 mutant channels showed a significant reduction in K^+^ current amplitude (Fig. 4b). CBD was seen to inhibit KCNT1 mutant currents with higher potency compared to WT KCNT1 (Fig. 4b). The IC_50_ for WT KCNT1 (5.6 ± 1.88 µM; (n = 4)) was significantly higher than IC_50_ for R398Q (0.41 ± 0.22 µM; (n = 4, *P* = 0.0436)) and R928C (0.47 ± 0.31 µM; (n = 4, *P* = 0.0458)) mutant channels (Fig. 4b). The difference between the IC_50_ for WT KCNT1 and G288S mutant channel (0.81 ± 0.34 µM), however, was not statistically different (n = 4, *P* = 0.0554). The time course of inhibition was similar between WT and mutant KCNT1 channels (Fig. 4c, data for WT and R928C KCNT1 are shown), and CBD was fully washable.

**Figure 5:**
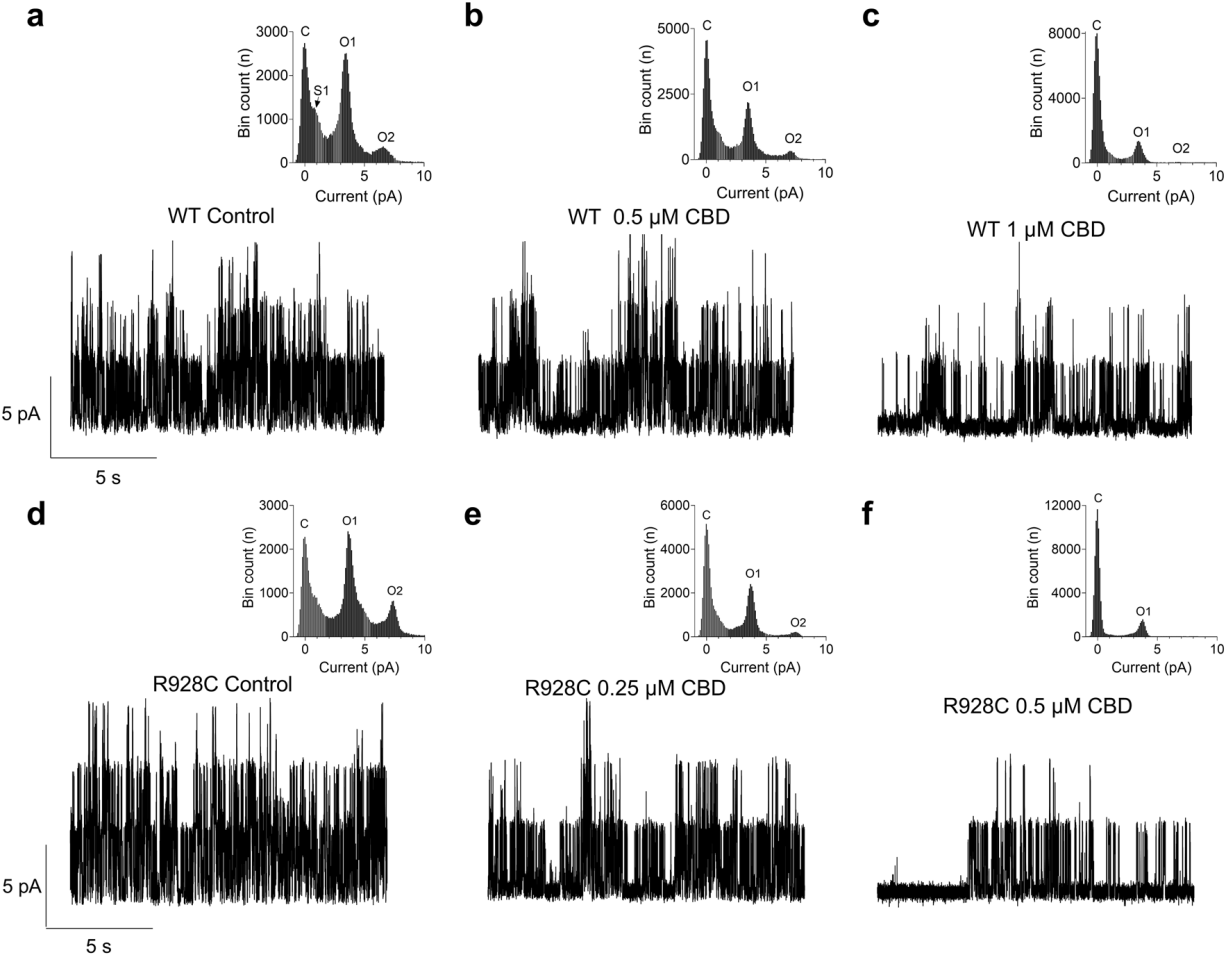
The effect of CBD on single WT and mutant (R928C) KCNT1 channels. Single WT (**a**–**c**) and mutant channel currents (**d**–**f**) were recorded at 0 mV in inside-out patches under control conditions and in the presence of the indicated amounts of CBD in the bath. The insets show the all-point amplitude histograms of the corresponding traces. C denotes the closed state, O1 and O2 are open states corresponding to the opening of one or two KCNT1 channels, whereas S1 is a substate.

Investigation of KCNT1 single channel activity using inside-out patches showed that CBD applied to the intracellular side of the membrane inhibited WT KCNT1 channels at lower concentrations, compared to the extracellular applications (Fig. 5a–c). Application of 0.5 µM CBD reduced *P*_O_ of WT KCNT1 by 17 ± 5% (n = 3) and 1 µM by 84 ± 11% (n = 3) (*c.f.* IC_50_ for extracellular application 5.6 ± 1.9 µM) without any effect on the single channel current (*i* = 3.6 ± 0.23 pA (control, n = 9) vs* i* = 3.7 ± 0.26 pA (0.5 µM CBD, n = 5) and *i* = 3.6 ± 0.34 pA (1 µM CBD, n = 5)). Similar to the extracellular applications, CBD inhibited R928C mutant more potently, compared to WT KCNT1, reducing *P*_O_ of R928C KCNT1 by 25 ± 5% (n = 3) at 0.25 µM and by 80 ± 7% (n = 3) at 0.5 µM (Fig. 5d–f). However, the difference between the efficacy of CBD inhibition of WT and mutant KCNT1 in inside-out patches was not as obvious as in the whole cell experiments.

### Responses of KCNT1 G228S, R938Q and R928C *Drosophila* seizure models to currently used drug treatments.

**Figure 6:**
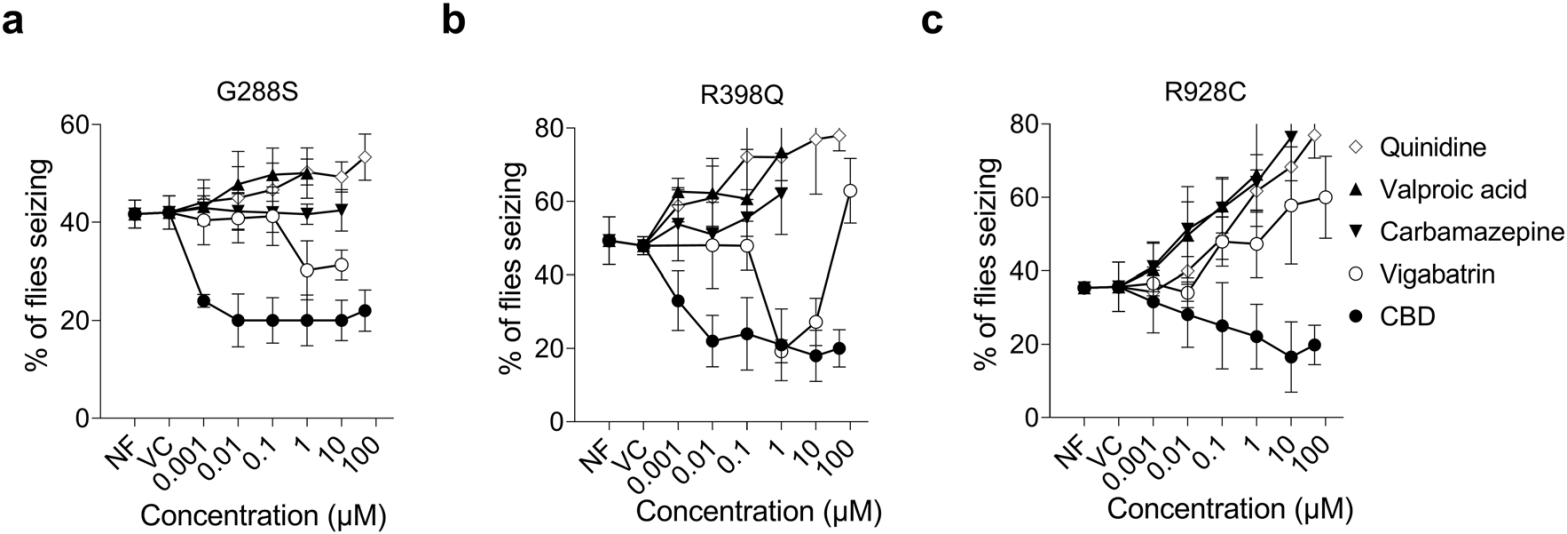
In vivo analysis of drug effects on seizure phenotype in *Drosophila* models. *Drosophila* expressing either G288S (**a**), R398Q (**b**) or R928C (**c**) mutant human *KCNT1* in GABAergic neurons were raised from embryos on normal food (NF) or on food containing a range of concentrations of CBD, vigabatrin, valproic acid, carbamazepine or quinidine and then analysed in the bang sensitive behavioural seizure assay. The percentage of *Drosophila* showing a seizure phenotype are shown for each dose of drug. All data points were compared to the vehicle control (VC) using Brown-Forsythe and Welsh’s one-way ANOVA followed by Dunnett's multiple comparisons test. Compared to vehicle control, CBD significantly reduced seizures in G288S and R398Q mutants at all concentrations; and R928C at 10 and 50 µM. Vigabatrin significantly decreased seizures in G288S and R398Q mutants at 1 and 10 µM, but increased seizures at 100 µM in R398Q mutant and R928C mutant at 10 and 100 µM. Valproic acid had no effect on G288S mutant, but increased seizures in R398Q at 1 µM and R928C at 1 and 10 µM. Carbamazepine had no effect on G288S and R398Q mutants, but significantly increased seizures in R928C at 0.1, 1 and 10 µM. Quinidine exacerbated seizures in G288S mutant at 50 µM, and R398Q and R928C mutants at 0.1, 1, 10 and 50 µM. The total numbers of flies, the number of independent crosses and the exact P values for all data points are presented in Supplemental Table 1.

We next investigated the effects of the drugs carbamazepine, valproic acid, vigabatrin, quinidine and CBD on the seizure phenotype of the three *Drosophila* lines expressing the mutant human *KCNT1* transgenes. We used feeding experiments with a range of drug concentrations, followed by the bang sensitive behavioural assay. To most closely match the human disease model of onset in infancy we fed *Drosophila* immediately from shortly after birth (*i.e.*, first instar larvae) through to just before assay for seizures 4–8 days post eclosion (hatching). None of the drugs were seen to have an effect on the viability or behaviour of *Drosophila* expressing WT *KCNT1* (data not shown). Carbamazepine, valproic acid and quinidine, were each seen to exacerbate the seizure phenotype in the three *KCNT1* mutant *Drosophila* lines (Fig. 6). Vigabatrin was seen to reduce the seizure phenotype in the lines expressing G288S and R398C mutant KCNT1, while it increased the seizure phenotype in the R928C mutant. CBD showed significant reduction of the seizure phenotype in all three *KCNT1* mutant lines and showed a dose-dependent response (Fig. 6). The maximum reduction of the seizures was 52% in the lines expressing G288S mutant (at 0.01–10 µM CBD); 63% in the lines expressing R398Q mutant (at 10 µM CBD); and 53% in the lines expressing R928C mutant (at 10 µM CBD). We also conducted adults only feeding of flies expressing R398Q KCNT1 to see if that was sufficient for rescue of seizures. Data for the rescue of *Drosophila* expressing R398Q human *KCNT1* mutant in GABAergic neurons only during adult stages (*i.e.,* post eclosion) is shown in Supplementary Fig. 2. While adult feeding also reduced seizures it was not as efficient as feeding from early larval stages, as they required higher doses of CBD (1uM and 10uM) to get significant amounts of rescue (Supplementary Fig. 2).

## Discussion

In this study we found that expression of human KCNT1 channels containing three patient-specific mutations gives a seizure phenotype in *Drosophila*. The phenotype was observed when the mutant channels were ectopically expressed in inhibitory GABAergic neurons. These results indicate that overactivity of the KCNT1 channel and increased K^+^ currents in inhibitory neurons is sufficient to induce a seizure phenotype in *Drosophila*. This finding is consistent with previous studies, including our recent analysis on the electrophysiological properties of a large series of patient *KCNT1* mutations^8^. KCNT1 channels are integral membrane proteins thought to be important in hyperpolarising the cell membrane, acting to inhibit excitability by reducing the likelihood of repetitive firing of action potentials. Greater silencing of inhibitory neurons by increased KCNT1 activity may lead to neuronal hyperexcitability, the mechanism that underlies seizures. Studies in mice also suggest that decreased excitability of inhibitory neurons contribute to seizures^13,14^. Expression of the three *KCNT1* mutants in all neurons, excitatory neurons or in glia in *Drosophila* were damaging, resulting in lethality. The reasons for this are not yet understood. The seizure phenotype of *KCNT1* R398C was significantly stronger than that seen with R928C. This may be consistent with R928C being only identified in patients with the “milder” phenotype of sleep-related hyper motor epilepsy (SHE), while R398C (and G288S) are also found in patients with more severe phenotypes including epilepsy of infancy with migrating focal seizures (EIMFS)^3^.

To ascertain whether our *KCNT1 Drosophila* models may be useful as tools for assessing drug treatments for *KCNT1*-epilepsy, we looked at the effects on the seizure phenotype for some of the drugs currently used to treat patients and reduce seizures in some patients. We also looked at the in vitro effects of the drugs in cell models expressing KCNT1 channels to see if findings from the two systems are comparable. Feeding vigabatrin, valproic acid and carbamazepine to *Drosophila* expressing WT KCNT1 channels showed no evidence of toxicity or alteration of normal behaviour. In the *KCNT1* mutant lines, valproic acid and carbamazepine, which are both known to inhibit voltage and use-dependent sodium channels^39^, were seen to exacerbate the seizure phenotype in a dose-dependent manner. Vigabatrin, which increases GABA levels by inhibiting GABA aminotransferase^40^, was seen to reduce the seizure phenotype at some doses in the animals expressing *KCNT1* G288S, and R398Q, and exacerbated the seizure phenotype in those expressing R928C. The seizure phenotype in the *Drosophila* models showed responses to the anti-epileptic drugs, with varied effects on the different mutant channels. Variable responses to the drugs are also seen in patients, with none being highly effective in inhibiting seizures. Further studies are needed to investigate if the *Drosophila* models will be useful in preclinical pharmacogenetics for predicting the response of patients with particular *KCNT1* mutations to different drugs.

Quinidine is a long-known blocker of ion channels and has previously been used as an anti-arrhythmic drug^41^. It has previously been shown in vitro to significantly reduce the K^+^ currents of WT^42^ and epilepsy-associated mutant KCNT1 channels^9,11,19^. However, it has shown variable results and serious side effects in some *KCNT1* epilepsy patients^3,16,19–21,43,44^. Given the conflicting in vitro and in vivo (patients) results, we analysed the effects of quinidine on *Drosophila* expressing the G288S, R398Q or R928C *KCNT1* mutants. The R928C and R398Q mutant models each showed a strong dose-dependent increase in the seizure phenotype, which was less pronounced in the G288S mutant, and no effect was seen in animals expressing WT *KCNT1*. The in vivo effects of quinidine on KCNT1 channels in this study therefore again differ from those seen in previous in vitro studies which show blocking of the channel activity. This is likely due to the presence, in vivo, of multiple interacting neural networks in the context of a functioning nervous system in a whole animal, and/or due to the inhibition of ion channels other than KCNT1 controlling the activity of the excitatory neurons, due to the higher concentrations of quinidine required to see the blocking effects in the in vitro studies^44^.

Ingestion of medicinal cannabis derivatives containing the compound cannabidiol (CBD) reduces the frequency of seizures in some *KCNT1*-epilepsy patients and has not been reported to exacerbate seizures^3,18^. Although the exact mechanism of action of CBD in epilepsy treatment is yet to be elucidated, it has been postulated that CBD modulates intracellular calcium and adenosine mediated signalling to inhibit neuronal activity^45,46^. In this work, CBD was seen to significantly reduce the K^+^ currents in HEK293T cells expressing WT and each of the G288S, R398Q, R298C mutant KCNT1 channels. The effect was rapid and at least similar or stronger in ‘inside-out’ patches suggesting CBD may be acting directly on the binding site in the channel, accessible from both sides of the membrane, rather than through cannabinoid receptor signalling. Significantly, CBD was approximately tenfold more efficient at blocking K^+^ currents of R398Q and R928C KCNT1 channels than WT KCNT1 channels. While this preferential action on mutant KCNT1 channels is a highly desirable property for a *KCNT1* therapeutic, it is unclear why this might be the case. It is possible that CBD binding to KCNT1 channel is state-dependent and the higher open probability of the mutant KCNT1 channels results in a higher efficacy of the drug^8^. This is supported by the results of the inside-out experiments where higher concentration of Na^+^ (35 mM) was employed to increase the *P*_O_ of WT channels. The dependence of GoF KCNT1 mutant channels on the intracellular Na^+^ is significantly shifted towards lower concentrations compared to WT channels^12^. As a result, with higher intracellular Na^+^ the difference in the *P*_O_ of WT and mutant KCNT1 channels is diminished, so is the difference in the CBD efficacy. While this is the first demonstration of direct inhibition of the KCNT1 K^+^ currents by CBD, further investigation into the mechanism of action on KCNT1 as well as the efficacy and dosing regimens for treatment of patients with CBD may also be warranted.

In summary, our study shows that the expression of patient-specific *KCNT1* mutations in *Drosophila* gives a seizure phenotype, modelling human *KCNT1*-epilepsy. The seizure phenotypes in the *Drosophila* models were affected by the addition of drugs currently used to treat people with *KCNT1* epilepsy, suggesting they may be useful as preclinical tools for screening new therapeutics for these epilepsies. Importantly, we were able to demonstrate differential effects, some positive and others negative, as well as differential efficacies, of currently used drugs. Together, the three *KCNT1* mutations investigated in this study account for approximately one fifth of patients identified to date, thus dosing information and any candidate drugs identified using the models will potentially benefit a significant proportion of people with *KCNT1* epilepsy.

## Materials and methods

### Generation of constructs and germ line transformation

Transgenic *Drosophila* were generated using the attP2 locus and PhiC31 integration system^47^. Full-length cDNA for Human *KCNT1* Transcript 1 NM_020822.1 (Origene Technologies Inc, Rockville, MD, USA) was mutagenized to induce point mutations, G288S, R398Q and R928C using QuikChange lightning site-directed mutagenesis kit (Agilent Technologies Inc. Santa Clara, CA, USA). Primers used for mutagenesis are as follows: p.G288S (5′-ACGGGGACCTGCAGCATCCAGCACC-3′ and 5′-GGTGCTGGATGCTGCAGGTCCCCGT-3′), p.R398Q (5′-CGCCCACCCCCAGCTCCAGGACT-3′ and 5′-AGTCCTGGAGCTGGGGGTGGGCG-3′) and p.R928C (5′-CCACCCTTCCAACATGTGCTTCATGCAGTTCCG-3′ and 5′-CGGAACTGCATGAAGCACATGTTGGAAGGGTGG-3′). The resulting constructs were cloned into EcoRI/XhoI restriction sites of pUAST-attB. All introduced mutations were verified by Sanger sequencing prior to injection into embryos (BestGene Inc. Chino Hills, CA, USA).

### *Drosophila* stocks and culture

*Drosophila* were cultured in 12-h light/dark cycles on a standard fortified medium containing 1% agar, 1% glucose, 6% fresh yeast, 9.3% molasses, 8.4% coarse semolina, 0.9% acid mix (4.6% orthophosphoric acid v/v, 43.9% propionic acid v/v Sigma-Aldrich (Gillingham, U.K.) and 1.7% Tegosept (Chem Supply, Australia). The following *Drosophila* lines were obtained from the Bloomington *Drosophila* Stock Center (Indiana, USA): W118 (BL3605), *elav*^*C155*^*-GAL4* (BL458), *GAD1-GAL4* (BL51630)*, Chat-GAL4* (BL56500) and *Repo-GAL4* (BL7415)*.* The ***GAD1-GAL4*** line BL51630 (created by Gero Miesenbock and deposited into Bloomington Drosophila Stock Centre by Prof Hugo Bellen) drives GAL4 expression with a 3.089 kb fragment of the GAD1 promoter, immediately adjacent from the translation start site. GAD1-GAL4 has been used numerous times by researchers to drive expression of UAS transgenes in GABAergic neurons^33–38^. The vector control BL8622 (y[1] w[67c23]; P(y[+ t7.7] = CaryP)attP2) was used to generate transgenic *Drosophila* with human *KCNT1.* Transgenic *Drosophila* harbouring human *KCNT1* generated in this work were: *UAS-KCNT1 WT*, *UAS-KCNT1 G288S*, *UAS-KCNT1 R398Q* and *UAS-KCNT1 R928C*.

### Bang-sensitive behavioural assays to investigate a seizure phenotype

Seizures were studied using the bang-sensitive behavioural assay which measures the occurrence of seizures induced by mechanical shocks^48^. All the experiments were performed between 8 and 11 am to minimize the effect of circadian rhythms on neuronal activity. Male and female flies aged between 4 to 8 days after eclosion were collected in batches of 10–20 flies under CO_2_ anaesthesia and transferred to a 100 ml (inner diameter: 29.5 mm) measuring cylinder (Cat# 612–3836, VWR International) where they were allowed to recover from anaesthesia and acclimatise for 5 min before they were assayed separately. Visibly unhealthy flies and those with damaged appendages were excluded from the assay.

Seizures in *Drosophila melanogaster* are characterised by a repertoire of behavioural abnormalities which include: an abnormal loss of posture, random wing flapping, leg shaking, spinning and uncontrolled flight, complete immobilization and falling down during uncoordinated flight and climbing efforts^27,49,50^.

For this study, animals were considered as normal if they were able to climb past a 5 cm mark on the cylinder after mechanical shock. Flies that did not climb past a 5 cm mark and exhibited immobilization but had a normal standing posture or were grooming themselves were also considered as normal. Flies were classified as displaying seizures if they were unable to climb past a 5 cm mark and showed one or more of the accepted seizure-like behaviours throughout 30 s post mechanical shock. The ‘seizure-like behaviours’ scored were: buzzing (fly was upside down at the bottom of the cylinder exhibiting continuous flapping of wings and legs), spinning (fly at the bottom erratically moving in circles), leg shaking (fly upside down continuously shaking its legs), uncontrolled flight attempts (attempted flight jump and then falling down to abnormal posture, recovery and then trying to fly), uncoordinated climbing effort (attempted climbing and then falling down and then again try to climb) and immobilized flies with an abnormal posture (bending sideways).

To look for the presence or absence of a seizure phenotype, flies were subjected to mechanical shocks by forcefully tapping the cylinder 20 times in “banging assays” which are a well-established method in investigating a seizure phenotype. A single researcher conducted the ‘banging’ and scoring for all experiments for intra-experimental consistency. To facilitate analysis and scoring the flies were videotaped during the process for 3 min with a Dino-Lite digital microscope (Product#AD3713TB; AnMo Electronics Corporation, New Taipei City, Taiwan). To determine the proportion of animals displaying seizure like activity, the resulting videos were zoomed in to cover up to the 5 cm mark on the cylinder. Individual flies were observed for a minimum of 30 s at a slower video playback speed (0.30X) to score seizure activity. In the banging assays a minimum of 50 Drosophila were analysed for each genotype with each concentration of a drug. The ≥ 50 flies were collected from a minimum of 3 independent crosses (N) and were individually analysed in measuring cylinders in groups of 10–20 adult flies per cylinder. Brown-Forsythe and Welch's ANOVA tests, followed by Dunnett’s multiple comparisons tests (GraphPad Prism 9) were used to determine the statistical difference of recovery from mechanical stimulation between different groups.

### Analysis of the effects of selected drugs on seizure activity in *Drosophila*

Based on clinical data, five of the frontline epilepsy drugs most commonly administered to patients with *KCNT1*-epilepsy. Drugs: carbamazepine (Sigma Aldrich: PHR1067), valproic acid (Sigma-Aldrich: P4543), vigabatrin (Sigma-Aldrich: V8261), quinidine (Sigma-Aldrich: R751839) and CBD (Gift from Professor Sanjay Garg) were analysed in vivo to determine their effects on the seizure phenotype of the three *Drosophila* lines expressing the mutant human *KCNT1* transgenes. We used feeding experiments with a range of drug concentrations, followed by the bang sensitive behavioural assay. Each drug was dissolved in absolute ethanol and a constant volume (1 µl) of 100% ethanol was dispensed per 1 ml of *Drosophila* food for all drug concentrations.

*Drosophila* were raised on standard fortified medium (composition mentioned above). *Drosophila* crosses were performed in cages with apple juice agar plates and yeast to encourage egg laying. Embryos were collected and transferred to vials with food containing the drugs at different concentrations (0.001–100 μM) at 24 °C. Adult *Drosophila* from these vials were allowed to age between 4 to 8 days on food containing the respective concentrations of drug to be tested. A range of concentrations of each drug dissolved in *Drosophila* food, between 0.001 μM and 100 μM, were used in the experiments. Multiple replicates were performed for each dosage of the drugs and controls. Controls were Vehicle Control (VC) which was the normal food with just the solvent ethanol present (1 μl/1 ml) in the *Drosophila* food, and Normal Food control (NF) where no drug or solvent (ethanol) was added in the *Drosophila* food. For Adult only feeding, R398Q mutant flies eclosed from normal food vials were allowed to age between 4–5 days on food vials containing CBD (0.1–10 μM) followed by the bang sensitive behavioural assay.

### Electrophysiology and analysis of effects of drugs

Whole-cell and inside-out patch-clamp recordings of KCNT1 mediated currents were performed using transiently transfected HEK293T cells 20–36 h post transfection. Whole-cell patch clamping was performed using a computer-based patch-clamp amplifier (EPC-9, HEKA Elektronik) and PULSE software (HEKA Elektronik) as previously described^8^. The bath solution contained 140 mM NaCl, 4 mM KCl, 2 mM CaCl_2_, 2 mM MgCl_2_ and 10 mM HEPES adjusted to pH 7.4 with NaOH. The pipette solution contained 80 mM K gluconate, 50 mM KCl, 10 mM NaCl, 1 mM MgATP, 10 mM EGTA and 10 mM HEPES adjusted to pH 7.3 with KOH. Patch pipettes were pulled from borosilicate glass and fire polished to give a pipette resistance between 1 and 2 MΩ. Series resistance ranged between 2.5 and 4 MΩ and was 80–90% compensated. For comparing amplitudes of KCNT1 currents produced by different constructs, the plasmids containing WT or mutant KCNT1 cDNA were transfected into HEK293T cells at the same amounts (0.8 µg in 35 mm Petri dish) and cells were used for patch clamping within a short time window 24–28 h post transfection. For the analysis of the drugs’ effects, the amounts of the plasmids and/or the time post transfection were adjusted to produce KCNT1 currents of similar amplitudes and amenable to voltage clamp with the voltage error less than 10% due to a residual uncompensated series resistance. The holding potential was set to − 78 mV and cells with the membrane potential more positive than − 72 mV were excluded from the analysis. Cell and the pipette capacitance were compensated for by the EPC-9 amplifier automatically. Expression of mutant KCNT1 channels had no effect on cell capacitance compared to cells expressing wild type KCNT1 (20.7 ± 7.4 pF (WT; n = 15) *vs* 19.9 ± 4.8 pF (G288S; n = 13), 21.0 ± 6.4 pF (R398Q; n = 14), or 20.9 ± 7.9 pF (R928C; n = 12)). Leakage through the seal determined immediately before achieving whole-cell configuration was subtracted using “leak subtraction” function of the EPC9 amplifier. No P/N protocol have been used due to KCNT1 channel non-zero open probability even at very negative potentials^8^. In the inside-out patch clamp experiments pipettes with a resistance between 2 and 4 MΩ were filled with a standard bath solution (see above) and the membrane patch was perfused from the intracellular side with a solution containing 110 mM KCl, 35 mM NaCl, 0.2 mM EGTA and 10 mM HEPES adjusted to pH 7.3 with KOH. Single channel current traces used for the analysis were recorded at 0 mV for the duration of 15 s, using 5 kHz sampling rate and 2 kHz filtering. For the display purposes all traces were further filtered by the 8-pole Bessel filter at 1 kHz. All drugs were dissolved in DMSO, aliquoted, and stored at − 20 °C. The maximum DMSO concentration in the bath solution did not exceed 0.05%. Drugs were applied using a gravity-fed perfusion system with the outlet positioned within 1–2 mm of the patched cell and the perfusion rate of 0.5 ml/min.

### Data analysis and statistics

Data were analysed using GraphPad Prism 9 software (San Diego, CA, USA). All values are reported as mean ± standard deviation (SD). All data passed normality and lognormality Shapiro–Wilk tests, determined by GraphPad Prism 9. In patch clamping data ‘n’ represents number of cells, and all experiments were repeated using cells from a minimum 2 separate transfections. To determine the IC_50_ of KCNT1 inhibition by CBD, the data were fitted with Hill equation of the form:

1 $${\text{Y}} = {\text{Bottom}} + ({\text{Top}} - {\text{Bottom}})/(1 + 10^{ \wedge } (({\text{LogIC}}_{50} - {\text{X}})*{\text{HillSlope}}))$$

where Top was constrained to 100% and Hill Slope to − 1.

Single channel data were analysed using Ana software developed by Dr Michael Pusch (Istituto di Biofisica, Genova, Italy) (http://users.ge.ibf.cnr.it/pusch/programs-mik.htm). The amplitude histograms generated by the Ana software were used to determine the changes in the open probability (*P*_O_) of KCNT1 channels. As most inside-out patches contained more than one active channel, typically 2–5, the *P*_O_ was calculated as 1-*P*_C_, where *P*_C_ is the probability of all channels in the patch being closed simultaneously. *P*_C_ was determined by dividing the bin count (equivalent to the area under the curve) corresponding to the closed state in the amplitude histogram by the total bin count of the entire histogram.

In statistical analysis of seizure-like behaviour of *Drosophila* (Supplementary Table) ‘N’ represents independent trials using independent fly crosses, with the total number of flies for each condition shown in brackets. In all experiments, statistical significance of differences between groups was determined using Brown-Forsythe and Welch's ANOVA tests, assuming non-equal SDs, followed by Dunnett’s multiple comparisons tests (GraphPad Prism 9).

### Supplementary Information

Supplementary Information.

## Data Availability

All data generated or analyzed during this study are included in this published article. Any materials, and data not included that support the findings of this study, are available upon reasonable request from the corresponding author. The DNA sequence of the full-length WT cDNA for Human *KCNT1* Transcript 1 can be found in the RefSeq database under the accession number NM_020822.1KCNT1. The DNA sequence of the KCNT1- G288S mutation can be found in the dbSNP database under RS number: dbSNP: rs587777264. The DNA sequence of the KCNT1- R398Q mutation can be found in the dbSNP database under RS number: dbSNP: rs397515407. The DNA sequence of the KCNT1- R928C mutation can be found in the dbSNP database under RS number: dbSNP: rs397515405.
