# Supplementary material for: Drosophila expressing mutant human KCNT1 transgenes make an effective tool for targeted drug screening in a whole animal model of KCNT1-epilepsy
Source: Sci Rep. 2024 Feb 9;14:3357. doi: 10.1038/s41598-024-53588-x (PMC10858247; doi:10.1038/s41598-024-53588-x)
Supplement: Supplementary file 1 — Supplementary Information. [file 41598_2024_53588_MOESM1_ESM.pdf]

Supplementary material

Drosophila expressing mutant human *KCNT1* transgenes make an effective tool for targeted drug screening in a whole animal model of KCNT1-epilepsy

Rashid Hussain[1], Chiao Xin Lim[1], Zeeshan Shaukat[1], Anowarul Islam[1,2], Emily A. Caseley[3], Jonathan D. Lippiat[3], Grigori Y. Rychkov[1,4,5], Michael G. Ricos[1], Leanne M. Dibbens[1*]

Affiliations:

[1]Epilepsy Research Group, Clinical and Health Sciences, Australian Centre for Precision Health, University of South Australia, Adelaide, South Australia 5000, Australia.

[2]College of Medicine and Public Health, Flinders University, Bedford Park, South Australia 5042, Australia

[3]School of Biomedical Sciences, Faculty of Biological Sciences, University of Leeds, Leeds LS2 9JT, UK

[4]School of Biomedicine, University of Adelaide, Adelaide, South Australia 5005, Australia

[5]South Australian Health and Medical Research Institute, Adelaide, South Australia 5005, Australia

*To whom correspondence should be addressed: Leanne.Dibbens@unisa.edu.au

Supplementary Table 1. P values obtained using One way ANOVA with Dunnett's multiple comparisons test of the data presented in Figure 6. Blue asterisks denote the statistically significant decrease in seizures compared to vehicle control, whereas red asterisks denote significant increase; ns – not significant. N is the number of independent fly crosses; number in brackets is the total number of flies analysed in each condition. The number of the control groups and the total number of flies for G288S, R398Q and R928C are 4 (54), 18 (314) and 4 (103) respectively.

	**G288S**					**R398Q**					**R928C**				
**[µM]**	CBD	Vigabtr	Valpr	Carbam	Quin	CBD	Vigabtr	Valpr	Carbam	Quin	CBD	Vigabtr	Valpr	Carbam	Quin
**0.001**	<0.0001<br>****<br>N=4(60)	0.9604<br>ns<br>N=4(60)	0.9999<br>ns<br>N=4(60)	0.9996<br>ns<br>N=3(62)	0.9979<br>ns<br>N=5(54)	0.0136<br>*<br>N=4(73)	0.1252<br>ns<br>N=5(101)	0.0526<br>ns<br>N=5(54)	0.6311<br>ns<br>N=5(77)	0.3140<br>ns<br>N=4(70)	0.9608<br>ns<br>N=4(73)	0.9998<br>ns<br>N=5(70)	0.8910<br>ns<br>N=5(54)	0.8675<br>ns<br>N=3(50)	0.9996<br>ns<br>N=4(67)
**0.01**	<0.0001<br>****<br>N=4(60)	0.9819<br>ns<br>N=4(60)	0.4985<br>ns<br>N=4(60)	0.9977<br>ns<br>N=3(60)	0.9758<br>ns<br>N=5(50)	<0.0001<br>****<br>N=4(50)	>0.9999<br>ns<br>N=4(50)	0.0613<br>ns<br>N=5(50)	0.9479<br>ns<br>N=5(74)	0.1634<br>ns<br>N=4(66)	0.5964<br>ns<br>N=4(52)	0.9996<br>ns<br>N=5(79)	0.0904<br>ns<br>N=5(50)	0.0550<br>ns<br>N=4(54)	0.8641<br>ns<br>N=4(51)
**0.1**	<0.0001<br>****<br>N=4(60)	0.9944<br>ns<br>N=4(60)	0.2154<br>ns<br>N=4(60)	0.9924<br>ns<br>N=4(55)	0.7269<br>ns<br>N=5(50)	<0.0001<br>****<br>N=6(158)	>0.9999<br>ns<br>N=4(76)	0.1073<br>ns<br>N=5(50)	0.4074<br>ns<br>N=5(51)	0.0012<br>**<br>N=5(54)	0.1644<br>ns<br>N=6(94)	0.2033<br>ns<br>N=4(74)	0.0037<br>**<br>N=5(50)	0.0023<br>**<br>N=5(55)	0.0125<br>*<br>N=6(58)
**1**	<0.0001<br>****<br>N=4(60)	0.0124<br>*<br>N=4(60)	0.1815<br>ns<br>N=4(60)	0.9997<br>ns<br>N=3(52)	0.1240<br>ns<br>N=4(55)	<0.0001<br>****<br>N=5(126)	<0.0001<br>****<br>N=5(132)	0.0006<br>***<br>N=5(68)	0.0569<br>ns<br>N=6(58)	0.0022<br>**<br>N=4(55)	0.0583<br>ns<br>N=5(91)	0.1963<br>**<br>N=5(99)	<0.0001<br>****<br>N=5(50)	0.0003<br>***<br>N=3(53)	<0.0001<br>****<br>N=5(51)
**10**	<0.0001<br>****<br>N=4(60)	0.0240<br>*<br>N=4(60)	0.9999<br>ns<br>N=4(60)	0.9996<br>ns<br>N=3(65)	0.2295<br>ns<br>N=5(50)	<0.0001<br>****<br>N=6(117)	0.0010<br>**<br>N=4(106)			0.0001<br>***<br>N=5(66)	0.0021<br>**<br>N=6(94)	0.0014<br>***<br>N=5(80)		<0.0001<br>****<br>N=5(67)	<0.0001<br>****<br>N=5(51)
**50**	<0.0001<br>****<br>N=4(60)				0.0153<br>*<br>N=5(50)	<0.0001<br>****<br>N=7(96)				<0.0001<br>****<br>N=5(59)	0.0096<br>**<br>N=7(86)				<0.0001<br>****<br>N=5(56)
**100**							0.0153<br>*<br>N=5(62)					0.0005<br>***<br>N=5(75)			

Supplementary Figure 1

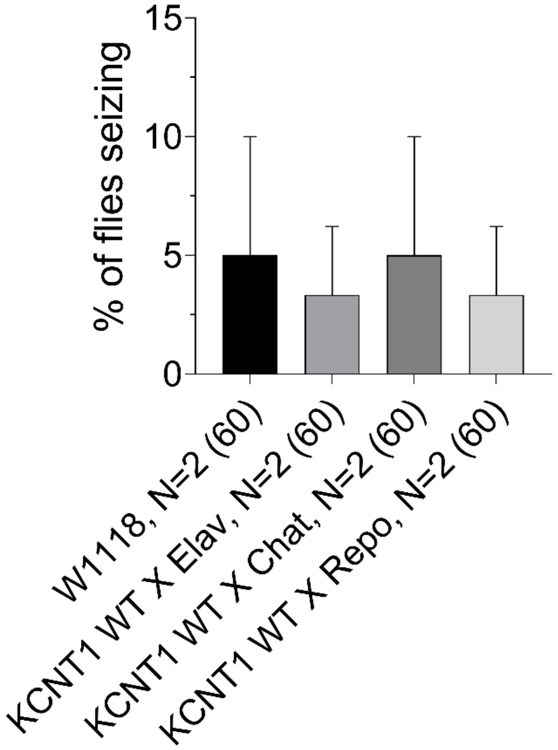

Figure S1. Expressing human *KCNT1* WT in different neurons of *Drosophila* gives no seizures in a bang sensitive behavioural assay. *Drosophila* with the vector control and WT human *KCNT1 e*xpressed pan-neuronal (*elav^{C155}-GAL4*), in excitatory neurons (*CHAT-GAL4*) and in glia (*Repo-GAL4*) were analysed in the bang sensitive behavioural seizure assay. Percentage of *Drosophila* showing a seizure phenotype are shown for each line. N, is the number of independent fly crosses, with total number of flies in all trials shown in the brackets. Brown-Forsythe and Welsh's one-way ANOVA followed by Dunnett's T3 multiple comparisons test showed no significant difference between the experimental groups.

Supplementary Figure 2

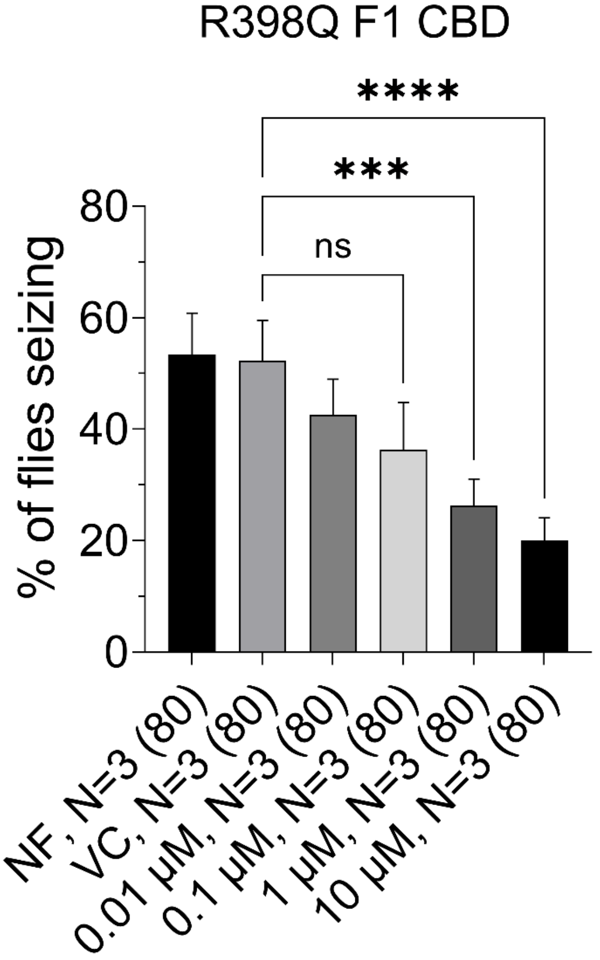

Figure S2. Feeding CBD to adult flies expressing human *KCNT1* mutant (R398Q) in GABAergic neurons of *Drosophila* rescues seizures in the bang sensitive behavioural assay. *Drosophila* expressing mutant human *KCNT1* with the R398Q mutation in GABAergic neurons were raised from embryos on normal food (NF) and the offspring were transferred on a food containing a range of concentrations of CBD (0.01-10 µM) and then analysed in the bang sensitive behavioural seizure assay. The percentage of *Drosophila* showing a seizure phenotype are shown for each dose of drug. N, is the number of independent fly crosses, with total number of flies in all trials shown in the brackets. All data points were compared to the vehicle control (VC) using Brown-Forsythe and Welsh's one-way ANOVA followed by Dunnett's multiple comparisons test. Compared to vehicle control, CBD significantly reduced seizures in R398Q mutants at 1 and 10 µM concentrations. **** P<0.0001, *** P=0.0006, and ns - no significant difference (P=0.0856 for 0.1uM).
